# The geography of hotspots of rarity-weighted richness of birds and their coverage by Natura 2000

**DOI:** 10.1371/journal.pone.0174179

**Published:** 2017-04-05

**Authors:** Fábio Suzart de Albuquerque, Andrew Gregory

**Affiliations:** 1 Science and Mathematics Faculty, College of Integrative Sciences and Arts, Arizona State University, Mesa, Azusa, United States of America; 2 School of Earth Environment and Society, Bowling Green State University, Bowling Green, Ohio, United States of America; Hungarian Academy of Sciences, HUNGARY

## Abstract

A major challenge for biogeographers and conservation planners is to identify where to best locate or distribute high-priority areas for conservation and to explore whether these areas are well represented by conservation actions such as protected areas (PAs). We aimed to identify high-priority areas for conservation, expressed as hotpots of rarity-weighted richness (HRR)–sites that efficiently represent species–for birds across EU countries, and to explore whether HRR are well represented by the Natura 2000 network. Natura 2000 is an evolving network of PAs that seeks to conserve biodiversity through the persistence of the most patrimonial species and habitats across Europe. This network includes Sites of Community Importance (SCI) and Special Areas of Conservation (SAC), where the latter regulated the designation of Special Protected Areas (SPA). Distribution maps for 416 bird species and complementarity-based approaches were used to map geographical patterns of rarity-weighted richness (RWR) and HRR for birds. We used species accumulation index to evaluate whether RWR was efficient surrogates to identify HRRs for birds. The results of our analysis support the proposition that prioritizing sites in order of RWR is a reliable way to identify sites that efficiently represent birds. HRRs were concentrated in the Mediterranean Basin and alpine and boreal biogeographical regions of northern Europe. The cells with high RWR values did not correspond to cells where Natura 2000 was present. We suggest that patterns of RWR could become a focus for conservation biogeography. Our analysis demonstrates that identifying HRR is a robust approach for prioritizing management actions, and reveals the need for more conservation actions, especially on HRR.

## Introduction

Although the natural rate of bird species loss is one species per century, in the last three decades 21 species have gone extinct, and currently 190 species worldwide are on the brink of extinction [1]. A key strategy to minimize species extinctions is to identify a group of sites that collectively represent all or most species in a small area. If the geographical distribution of individual species is known for most locations in a planning area, efficient sets of sites can be identified by spatial prioritization algorithms such as integer programming [2], by heuristic reserve-selection algorithms such as Marxan [3], C-Plan [4], Zonation [5], or by rarity-weighted richness (RWR, [6]). RWR is used to identify sites with both a relative rarity of species and overall richness [7]. RWR ranks sites from 0 (all species can be represented without the site) to +∞ (the site is indispensable to the goal of representing all species). These ranks reflect complementarity, i.e., how well the accumulation of sites jointly represents common and rare species with a small number of sites [8]. Stein et al [7] used biogeographical patterns of RWR to identify hotspots of rarity-weighted richness (HRR) across the United States. They concluded that identification of HRR has become an important tool for implementing conservation actions to selected areas (e.g. conserve imperiled species). Csuti et al. [9] and Albuquerque & Beier [8] provided comparisons of RWR solutions to solutions produced by richness, linear programing and simulated annealing (one of the most effective algorithms to identify priority areas for conservation) and observed that RWR represented biodiversity almost as effectively as sites identified by linear programing, and was more effective than species richness and simulated annealing approaches. Albuquerque and Beier [8] also suggested that RWR is suitable for prioritizing large datasets.

By contemplating conservation actions, biogeographers, conservation planners and stakeholders are often concerned with saving species from extinction and maintaining biodiversity [10]. Usual conservation actions, such as the establishment and management of protected areas (PAs), have helped to achieve the long-term conservation of nature, ecosystem services and cultural values [11]. PAs can also help to reduce pressure from human activities (e.g. anthropogenic transformation of the ecosystems), especially at areas located in intensively human disturbed regions [12]. A good example of PAs network is the European network of protected areas (Natura 2000, [13]). The Natura 2000 network aims to promote conservation and sustainable use of natural resources in continental and marine areas across 27 European Union (EU) countries. Natura 2000 is often considered as one of the single most important conservation tools in Europe and is crucial to ensure the survival of Europe’s most valuable species and habitats [13]. Natura 2000 network includes: areas under the Habitats Directive [14]–including Sites of Community Importance (SCI) and Special Areas of Conservation (SAC)—and sites under the Birds Directive [15]–including Special Protected Areas (SPA). The former seeks to conserve biodiversity through the persistence of most the patrimonial species and habitats across Europe, whereas SPA aim to protect and conserve wild birds naturally occurring in EU countries [13]. Both directives are regarded as a basis for nature conservation throughout Europe.

The success of nature conservation actions is generally measured in terms of species richness and in particular bird species richness [16]. Until recently, few maps featuring equal area grid cells that harbor the largest numbers of terrestrial birds, endangered terrestrial birds or range-restricted terrestrial birds were produced in Europe (e.g. [17,18,19,20,21]). However, species richness could be a poor indicator of bird diversity because richness does not reflect complementarity. When sites with high species richness contain overlapping assemblages of species, a collection of species-rich sites fails to represent species efficiently [22]. Alternatively, rarity weighted richness and the distribution of hotspots of rarity-weighted richness may be a better predictor of conservation priority, because HRR takes into account not only number of species and proportional abundance of species presence, but also the occurrence of rare species of special conservation priority [8].

The present study aims to: 1) describe patterns of RWR and identify HRR for bird species across EU countries, 2) to describe the anthropogenic transformation caused by human interaction within each HRR, 3) to explore whether HRR are well represented by the Natura 2000 network, 4) to identify the most suitable SCI/SCA sites to designate them as SPAs. The specific goals were: 1) to describe the geographic distribution of RWR values and HRR of bird species in EU countries, 2) to evaluate the performance of species richness and RWR as surrogates of bird diversity, 3) to identify whether the spatial coverage of SPA and SCI areas follow the RWR pattern, and 4) to identify gaps of bird conservation. Our experiments with RWR are intended as theoretical tests of its effectiveness of RWR as a surrogate; the sites selected in our analyses are not intended to represent conservation priorities.

## Methods

### Data preparation

Distribution data for 495 European bird species were obtained from European Bird Census Council Atlas of European Breeding Birds (EBCC) [17]. These data represent 25 years of intensive field surveys by ornithologists in more than 40 countries [17]. Of the original set of species, a total of 416 bird species found in EU countries were selected. The presence of these species was recorded in each cell of an UTM grid comprising 1,695 cells of 50 × 50 km. RWR was calculated for each 50 × 50 km cell following the methods of [8].

Within the analysis, we classified the rarity of birds in two ways. First, bird species were classified according to the Birds Directive [15] as important bird species (IBS). IBS birds are defined by the Bird Directive as important bird species that shall be subject of species conservation measures to safeguard their survival and reproduction (species listed in the Bird Directive Annex I) (139 species). Secondly, birds were also classified according to the Red List categories as endangered (EN, 4 species), vulnerable (VU, 15 species), near threatened (NT, 22 species) and least concern (LC, 361 species). Fourteen species were not assessed (NA) in the IUCN [23] database.

We obtained shapefiles and rasters depicting the known distribution of Natura 2000 [24] and of Anthropogenic Biomes of the World [25]. We processed these maps in grass 6.4.2 [26] to calculate the percentage of SPA cells and combined PAs (including SPA, Sites of Community Importance and Special Areas of Conservation) and to calculate the percentage anthropogenic transformations with each grid cell. A cell was considered protected, and bird species associated with that cell were considered represented, if any portion of the cell was protected. Otherwise the cell was considered unprotected. This procedure overestimates the degree to which Natura 2000 sites cover cells containing each bird species, and makes our analysis of the degree to which Natura 2000 fails to cover species of conservation concern a conservative estimate. The Anthropogenic Biomes of the World describes the anthropogenic transformations caused by sustained direct human interaction with ecosystems and it is based on population density, land use and vegetation cover [25].

### Estimating rarity-weighted richness

The rarity-weighted richness index [6] was used to identify the relative rarity of species in each cell. The RWR was calculated in two steps: (1) each species was scored as the inverse of the number of sites where it occurs. Thus, if a species is found in only in one site, the species receives the maximum score (1.0), whereas a species that occurs in 100 sites receives a lower score (1/100 or 0.01); and (2) the RWR score for each cell is the sum of individual scores of all species occurring in this cell:

$$RWR=\overset{n}{\underset{1}{\sum }}\frac{1}{c_{i}}$$

where *c*_*i*_ is the number of sites occupied by species *i*, and the values are summed for the *n* species that occur in that site.

Pearson's correlation was used to relate RWR values with PA cover (SPA and combined Natura 2000 areas). To explore the potential influence of spatial autocorrelation on Pearson's correlation, a modified *t* test proposed by Dutilleul [27] was used to check for overall consistency of statistical power of the correlations.

### Evaluating performance of RWR as a surrogate for bird species representation

Species richness and RWR were each evaluated in terms of their ability to identify cells that represented many species in relatively few cells. For each surrogate, cells were selected starting with the cell with the highest richness or RWR and adding the cell with the next highest richness/RWR at each succeeding step. At each step, the number of species represented in at least one cell of the hypothetical reserve was calculated.

The Species Accumulated Index (SAI, [28]) was used to evaluate the efficiency of species richness and the RWR in representing bird species. SAI compares *S*, the number of species represented in the set of sites selected using the RWR, to an optimum or near-optimum value *O* (the largest number of species that can be represented in the same number of sites) and to *R*, the mean number of species represented in the same number of randomly selected sites. To calculate *S*, cells were accumulated starting with the cell with the highest richness or RWR, and gradually adding the cell with the next highest value. As cells were accumulated, the number of species represented in at least one cell was calculated.

The core area version of Zonation [5] was used to calculate *O*. Zonation starts with all cells tentatively ‘reserved’ and iteratively removes cells that are least needed to maintain core areas of each species. As cells are progressively removed, this rule tends to remove cells that contain only widespread species (species with many cells remaining in the tentative solution); thus solutions tend to conserve “core areas” of roughly equal number of cells for all species. This produces a hierarchy of cells, accounting for complementarity [29]. Cells receive a rank between 0 and 1 –where values close to 1 indicate cells removed in the last step of the process, whereas values close to 0 indicate cells removed early (see Moinlanen et al. [29] for further details).

To calculate the number of species represented in the same number of randomly selected sites (*R*), cells were accumulated in random order and at each step; the number of species represented at least once in the randomly selected cells was calculated. This random procedure was repeated 1,000 times to calculate a mean value of R and a 95% CI on *R*.

The SAI was defined as the ratio SAI = (*S-R*)/(*O-R*). SAI is scaled –∞ to +∞; negative SAI indicates a worse than random result, 0 indicates random performance, and positive SAI is a measure of efficiency. For example, a SAI of 1 would indicate that RWR is as effective as Zonation (and therefore a reasonable estimate of an optimal solution). SAI was calculated at 15%, 20%, 25%, 30%, 35%, 40% and 45% of the numbers of cells hypothetically selected. The mean of these 7 SAI values was used as an overall estimate of RWR performance.

### Identifying Hot Spots of Rarity and Richness (HRR)

Cells within the top 15% (254 cells) of ranked RWR values were considered HRR, the most efficient reserve network for representing birds, ignoring connectivity and variation in cost of conserving land. This percentage reflects the proportion of land preserved for environmental conservation in Europe [30,31].

HRR without Natura 2000 cover were considered as total gap cells, and HRR outside SPAs but inside SAC/SCIs were considered as gaps of SPAs.

Analyses were performed in R [32] Geographic Resources Analysis Support System (GRASS GIS 6.4 [26]).

## Results

**Fig 1:**
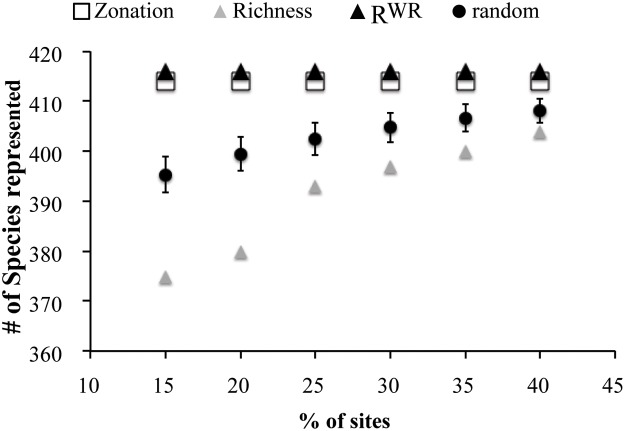
Number of species represented in sites selected in order of rarity-weighted richness (solid black triangles), by species richness (solid grey triangles) and by Zonation (open cells). The random solution and the 95% confidence interval (in black) are also shown.

For all percentages of sites prioritized, Zonation and RWR solutions performed better than randomly selected sites (Fig 1). The mean SAI across percentage of species prioritized was 1.28, indicating that RWR solutions were better (closer to the true optimum) than Zonation. Species richness was a poor surrogate for HRR, as indicated by the species accumulation index (Fig 1).

**Fig 2:**
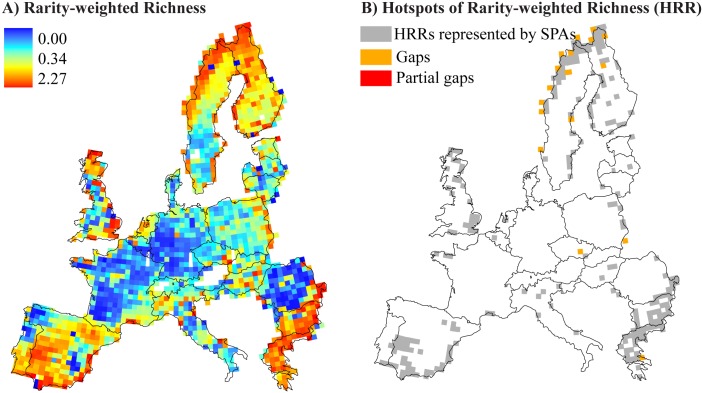
Geographical patterns of (A) Rarity-Weighted Richness (RWR) in EU countries and (B) Hot Spots of Rarity and Richness (HRR). Grey cells represent HRR covered by Natura 2000 network, orange cells represent HRR covered by SCA/SCIs only (partial gaps) and red cells represent HRR not covered by any Natura 2000 (gaps).

The geographical patterns of RWR showed no clear trends in EU countries. Higher RWR values were recorded in Finland, Great Britain, the Balkans and Iberian Peninsula. Regions with low RWR values occurred in central and southeast Europe (Fig 2A). Pearson’s correlations between birds RWR and the cover of PA in Europe (SPA and combined Natura 2000 cover) were in general weak (Pearson's r = +0.22 for SPA and r = +0.29 for all Natura 2000 protected areas) (Table 1).

**Table 1:** Correlations of rarity-weighted richness with Special Protected Areas (SPA) and combined protected areas cover (combined), including SPA, Sites of Community Importance and Special Areas of Conservation. Significance estimates are corrected for spatial autocorrelation using modified *t*-test [27]. The number of grid cells used in the analysis (N), corrected degree of freedom (D.F.) and the corrected probability are also given.

Natura 2000 areas	Pearson's r	N	D.F.	Probability
SPA	0.22	1695	680	<0.001
Combined	0.29	1695	352	<0.001

**Fig 3:**
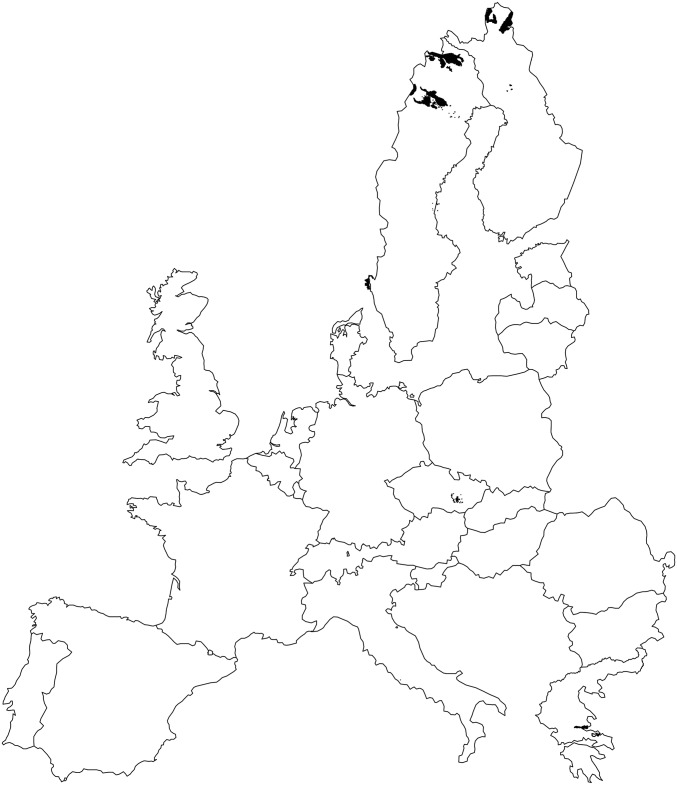
Geographical distribution of areas under Habitat Directive across partial gaps in EU countries.

Of the 254 HRR cells, 18 (7%) were not represented by SPA. From these, 11 were represented by SAC/SCI but not SPA (partial gaps), and 7 cells were not represented by Natura 2000 (gap cells). Most partial gap and gap cells were located in northern Europe (particularly in Finland and Sweden), although partial gaps were also present in localized areas of southern Czech Republic and Greece (Fig 2B). The 18 cells not represented by SPA included 307 (74%) of bird species, including 83 Important Bird Species, according to the Birds Directive (Directive 2009/147/EC). Of all not included species, 8 were considered vulnerable and 14 were considered near threatened. Partial gaps included 84 areas under Habitat Directive (SAC/SCI). Although, the highest number of these areas were observed in Czech Republic (47) and Sweden (28) (Fig 3), 95% of the SCA/SCI area were located in Sweden (68%) and Finland (27%). A list with a complete name and location of SCA/SCI areas is in the S1 Table.

**Fig 4:**
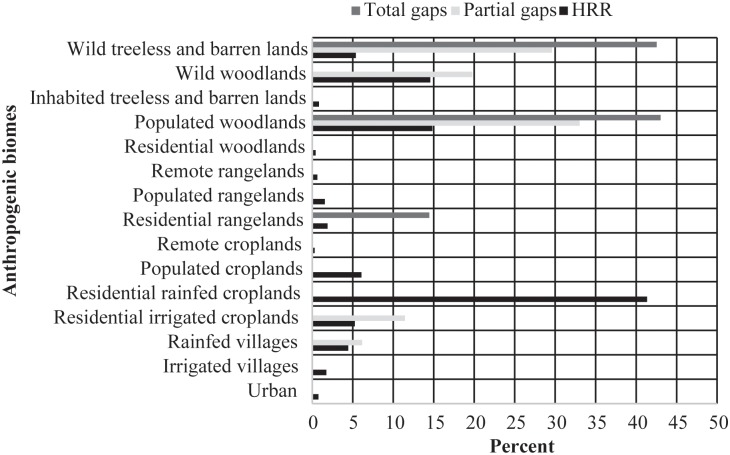
Percentage of anthropogenic biomes in Hot Spots of Rarity and Richness (HRR), partial gaps and total gaps.

Residential rainfed croplands, representing the mix of trees and rainfed cropland with substantial human population, were the most extensive of the populated biomes, covering nearly 42% of HRR area (Fig 4). After rainfed croplands, populated woodlands and wild woodlands were the second most extensive of the anthropogenic biomes. Wild treeless and barren lands and populated woodlands were the most common in partial and total gaps, covering 30 and 43% of cells area (Fig 4).

## Discussion

This study investigated the relationship between the calculated rarity-weighted richness values for 50 x 50km cells in EU countries and the Natura 2000 coverage and identified Hot Spots of Rarity and Richness for birds by assessing the biogeographical distribution of RWR values. This study does not aim to criticize the effectiveness of the Natura 2000 network in reducing bird diversity loss in the Europe, but rather to provide a robust analysis of the importance of the Natura 2000 network in representing HRR in this region. Because a cell was tallied as protected if any portion of the cell was overlapped by SPAs or SCIs, the analyses may overestimate how well SPAs and Natura 2000 sites represent bird species.

Residential rainfed croplands was remarkably the most extensive of the populated biomes found across HRRs. These may be associated with some HRR in eastern Poland, southern Romania and Bulgaria. Agricultural-dominated landscapes are very common in the continent, however, high proportion of rural areas among HRR in Europe could be also to the loss of biodiversity in agricultural landscapes in many states of the EU countries (e.g. farmland birds in western EU states) [33]. In the last 30 years, many common species have become rare or have disappeared because of more intensive forms of agricultural production [34, 35] and socioeconomic changes to human settlements in rural areas [36]. To avoid the significant decline of species in agricultural lands, experts suggest major changes in policy based on balanced technology and scientific knowledge [35]. Depending of the way agricultural areas are managed, it is possible to sustain high levels of wild native biodiversity [35, 37, 38].

This study provides new information about trends of birds RWR values patterns across the EU. Despite the Mediterranean basin, this study identified high RWR values across northern Europe (alpine and boreal biogeographical regions). The results suggest two non-exclusive reasons for the large number of HRR in this region. First, alpine environments have a great variety of ecosystems and habitats types observed in northern Europe, a high fauna and flora diversity and a high level of endemism [39]; and therefore, provide many areas to concentrate relatively rarity and overall richness. Second, the alpine region may be a potential refuge for animal and plants with large area requirements [39]. Refuges are considered resource-rich areas that are critical for the persistence of many species [40]. The alpine regions of northern Europe are relatively undeveloped and so may represent one of the last remaining large refuges left in Europe [41]. The sites selected in our analysis are not intended to maintain the entire bird diversity in Europe. For such analyses, important factors such as histories of life, home ranges, should be considered.

In a World of limited data on species distributions, RWR could be a useful metric to meet part of the Natura 2000 objectives. First, RWR can efficiently represent all birds in a small number of sites. Second, RWR can identify a set of priority areas with high concentration of range-restricted species, and high species richness. Third, RWR can highlight several other regions that rank highly based on the combination of rare and common species [7]. The survival of these species may require protection in multiple locations. Fourth, RWR can be used to extend the existing protected area network. Because actions to maximize the efficiency of PAs network at protecting diversity are urgently needed, RWR may assess the contribution that each site makes to overall protection goals [7].

The weak association between RWR values pattern and Natura 2000 cover (Table 1) suggests that the design of the Natura 2000 network is not accounting for spatial patterns calculated from quantitative-based approaches, such as RWR. This result also may support the preposition that the establishment PAs often relies on socio-political and economical concerns other than conservation goals [42]. This does not mean that the Natrura 2000 network is not functional, since this network represents all species. However, a quantitative-based approach helps to design a cost-efficient network of priority areas—a comprehensive, representative, and adequate for maximizing the representativeness of the full biological diversity of a region with a minimum number of sites [43]. In addition, quantitative-based methods have been extensively used to guide management decisions that try to safeguard natural processes affecting the distribution of biodiversity [5]. This result, however, could be masked by the geographic coverage of this study, since PAs are not always designed to protect bird species, or with the same scale as gradients of species distribution, which may be a limitation when assessing the efficiency of Natura 2000. In addition, the spatial scale of the PAs may not be in accordance with the scale at which bird species range distribution is measured. Even with these limitations, most of the birds occurring in the EU are migratory species that require trans-frontiers actions for an effective representation [5]. To be effective, EU countries must address large-scale and iterative approaches into conservation policies.

Natura 2000 is designed to achieve the long-term conservation of nature and ecosystem services [11], but its effectiveness has been questioned [8,44]. There is no doubt that SPAs have averted declines and improved prospects for many bird species in Europe [45]. This study indicates that Natura 2000 cells protect large core areas (HRR) for many species. However, 18 HRR were not represented by SPAs. If some SACs are also designated as SPAs, an additional 11 HRR would be covered, and several unthreatened and threatened species would be better protected. Furthermore, SPAs bring substantial conservation benefits to bird populations [46], and also establishes a general policy to avoid bird kill or capture, destruction of eggs, and nest disturbance [20]. The grain of analysis however could affect this result, since the cell site is much larger than the spatial resolution at which sites are prioritized for conservation.

Results showed a poor performance of species richness in maximum bird species representation. In all cases, cells ranked with high species richness represented less species than the random solution. The poor job of the richness solution may be because areas with high richness are quite similar to each other in terms of species composition and also because unique or rare species may occur in areas of low richness [43]. The utility of richness as a tool for prioritizing land for conservation was also questioned in previous studies [47, 48]. On the other hand, results indicated that the number of species in sites assembled in RWR rank order can be used as an approximation of the maximum number of species that can be represented in a given number of sites. Results also indicated that RWR was as efficient than the Zonation framework to represent bird diversity. Algorithms like simulated annealing and Zonation often produce optimum or near-optimum solutions [49]. Because RWR was more efficient than Zonation and simulated annealing [9], RWR must also be producing optimum or near-optimum solution in Europe. In Addition, RWR represented 100% of the total number of bird species at all percentages of sites selected, leaving little room for superior solutions. This result gives support to the notion that RWR is a reliable alternative to heuristic algorithms for identify the minimum areas needed to protect species [7,9].

In summary, the results of this study have three important implications for conservation of bird diversity in Europe. First, the Mediterranean Basin and the alpine and boreal biogeographical regions of northern Europe emerge as the hotspots of rarity and richness for bird species in Europe. Both regions have a high number of endemic and threatened species. Thus, urgent conservation actions are required to halt biodiversity loss in those areas. Second, residential rainfed croplands were the most frequent biomes among the HRR indicating the importance of saving biodiversity of agricultural landscapes in Europe. Third, Natura 2000 coverage did not closely match bird RWR values, indicating that the establishment of PAs across Europe may not be fully accounting for RWR patterns. It would be useful to analyze a dataset with the same extent with fine grain data (e.g. 100m x 100m). Unfortunately, there is limited data available for this grain. Natura 2000 is probably the most powerful mechanism to ensure protection of HRR, but funding is limited and opportunity costs of conservation can be large. The spatial distribution of HRR and gaps of bird conservation showed herein may provide an efficient way to expand the Natura 2000 network across Europe.

## Supporting information

S1 Table List of areas under Habitats Directive located in Hot spots of rarity-weighted richness RWR in European Union. (DOCX)
