# Supplementary material for: The geography of hotspots of rarity-weighted richness of birds and their coverage by Natura 2000
Source: PLoS One. 2017 Apr 5;12(4):e0174179. doi: 10.1371/journal.pone.0174179 (PMC5381779; doi:10.1371/journal.pone.0174179)
Supplement: S1 Table — (DOCX) [file pone.0174179.s001.docx]

**Supporting information**

**S1 Table – List of areas under Habitats Directive located in Hot spots of rarity-weighted richness RWR in European Union.**

| SITECODE | SITENAME | Country | Area (m2) |
| --- | --- | --- | --- |
| CZ0620051 | Slapanicke slepence | Czech Republic | 83239.54404 |
| CZ0620120 | Zlobice | Czech Republic | 615859.3371 |
| CZ0620132 | Udoli Chlebskeho potoka | Czech Republic | 1369836.243 |
| CZ0622173 | Netopyrky | Czech Republic | 9128.455777 |
| CZ0622217 | Stepni strane u Komoran | Czech Republic | 118776.2845 |
| CZ0623704 | Dlouha Lhota | Czech Republic | 372.622156 |
| CZ0623713 | Rosice - zamek | Czech Republic | 2343.679486 |
| CZ0623775 | Bucovice - zamek | Czech Republic | 3213.972183 |
| CZ0624065 | Kvetnice | Czech Republic | 1275314.306 |
| CZ0624094 | Bosonozsky hajek | Czech Republic | 481048.964 |
| CZ0624132 | Udoli Svitavy | Czech Republic | 12048184.76 |
| CZ0713388 | Protivanov | Czech Republic | 23013.29793 |
| CZ0713723 | Cechy pod Kosirem | Czech Republic | 3931.83036 |
| CZ0620245 | Rakovecke udoli | Czech Republic | 7558074.886 |
| CZ0612133 | Dedkovo | Czech Republic | 56347.0686 |
| CZ0622170 | Na lesni horce | Czech Republic | 25953.68473 |
| CZ0622174 | Pansky les - Jezdiny | Czech Republic | 260870.8972 |
| CZ0622220 | Bila hora | Czech Republic | 17871.56189 |
| CZ0623025 | Slavkovsky zamecky park a aleje | Czech Republic | 212689.49 |
| CZ0623329 | Prudka | Czech Republic | 1385.42534 |
| CZ0623344 | Nad Brnenskou prehradou | Czech Republic | 5671687.515 |
| CZ0623354 | Crhov - Rozsicka | Czech Republic | 274933.9838 |
| CZ0623701 | Blansko - kostel | Czech Republic | 704.0532826 |
| CZ0623702 | Borotin - zamek | Czech Republic | 1174.368375 |
| CZ0623709 | Kretin - zamek | Czech Republic | 948.0879171 |
| CZ0624020 | Stranska skala | Czech Republic | 168046.8917 |
| CZ0624062 | Cernecky a Milonicky hajek | Czech Republic | 2040759.476 |
| CZ0624067 | Kamenny vrch | Czech Republic | 137777.9369 |
| CZ0624235 | Malhostovicke kopecky | Czech Republic | 26343.86742 |
| CZ0624236 | Jizni svahy Hadu | Czech Republic | 299003.6041 |
| CZ0712225 | Za hrncirkou | Czech Republic | 31106.7304 |
| CZ0713383 | Ohrozim - Horka | Czech Republic | 921.0808876 |
| CZ0620194 | Cepickuv vrch a udoli Hodoninky | Czech Republic | 1874705.54 |
| CZ0620002 | Clupy | Czech Republic | 180485.574 |
| CZ0623710 | Krtiny - kostel | Czech Republic | 1967.393148 |
| CZ0712191 | Stran nad Hutskym potokem | Czech Republic | 8038.397095 |
| CZ0620191 | Sokoli skala | Czech Republic | 3051560.721 |
| CZ0620018 | Vetrniky | Czech Republic | 323598.4714 |
| CZ0620037 | Sivicky les | Czech Republic | 2365961.855 |
| CZ0623351 | Nad kaplickou | Czech Republic | 38360.41794 |
| CZ0623366 | Strelicka bazinka | Czech Republic | 29273.72353 |
| CZ0623370 | Letiste Marchanice | Czech Republic | 208789.9281 |
| CZ0623703 | Dedice - kostel | Czech Republic | 600.4173335 |
| CZ0623807 | Hobrtenky | Czech Republic | 1311897.327 |
| CZ0623808 | Pisarky | Czech Republic | 707085.1975 |
| CZ0624129 | Lucni udoli | Czech Republic | 1259984.652 |
| CZ0624130 | Moravsky kras | Czech Republic | 64866088.59 |
| FI1300212 | INARIJÄRVI | Finland | 899575233.3 |
| FI1301316 | Herankaira | Finland | 22172671.52 |
| FI1302003 | Paistunturin erämaa | Finland | 1597678118 |
| FI1300204 | Vätsärin erämaa | Finland | 1573518643 |
| FI1301313 | Narkauksen-Katiskon lehdot | Finland | 938013.997 |
| FI1301304 | KAIHUAVAARAN LEHTO | Finland | 133634.7117 |
| FI1301312 | NAMALIKKOKIVALO | Finland | 8359909.484 |
| GR2410001 | LIMNES YLIKI KAI PARALIMNI - SYSTIMA VOIOTIKOU KIFISOU | Greece | 116144166.4 |
| GR2440002 | KOILADA KAI EKVOLES SPERCHEIOU - MALIAKOS KOLPOS | Greece | 475643079.3 |
| SE0520170 | Kosterfjorden-Väderöfjorden | Sweden | 539896960 |
| SE0630171 | Gnarpskaten | Sweden | 1504202.8 |
| SE0520172 | Idefjorden | Sweden | 8802381.341 |
| SE0630170 | Vitörarna | Sweden | 1542569.481 |
| SE0820129 | Daita | Sweden | 400231.2026 |
| SE0820130 | Udtja | Sweden | 1464878772 |
| SE0820282 | Torneträsk-Soppero fjällurskog | Sweden | 3366356014 |
| SE0820293 | Norra Torneträsk | Sweden | 458923123.9 |
| SE0820126 | Långsjön-Gåbrek fjällurskog | Sweden | 72678923.7 |
| SE0820261 | Abisko | Sweden | 77223678.33 |
| SE0630251 | Oppegården | Sweden | 30270.2035 |
| SE0820434 | Piteälven | Sweden | 48762454.17 |
| SE0630173 | Gran | Sweden | 4739960.61 |
| SE0630227 | Klovbäcken | Sweden | 49353.24415 |
| SE0820123 | Hornavan-Sädvajaure fjällurskog | Sweden | 804539689.6 |
| SE0820156 | Pärlälvens fjällurskog | Sweden | 1157619472 |
| SE0820334 | Sulitelma | Sweden | 619105479.4 |
| SE0820621 | Låktatjåkka | Sweden | 75800445.55 |
| SE0820623 | Nissuntjårro | Sweden | 257719664.9 |
| SE0630093 | Norra Hornslandet | Sweden | 1116006.389 |
| SE0630111 | Bromsvallsberget | Sweden | 191729.4621 |
| SE0630172 | Gnarps Masugn | Sweden | 2257443.808 |
| SE0630182 | Oxsand | Sweden | 303562.6823 |
| SE0820074 | Ståkke-Bårgå fjällurskog | Sweden | 220555658.9 |
| SE0820124 | Tjeggelvas | Sweden | 328238750.3 |
| SE0820125 | Ramanj | Sweden | 46441283.95 |
| SE0820234 | Stordalen | Sweden | 11351350.6 |
| SE0820243 | Rautas | Sweden | 816698092.1 |
